# Response: Commentary: Multimodal theories of recognition and their relation to Molyneux's question

**DOI:** 10.3389/fpsyg.2016.00173

**Published:** 2016-02-12

**Authors:** Nicholas Altieri

**Affiliations:** Communication Sciences and Disorders, ISU Multimodal Language Processing Lab, Idaho State UniversityPocatello, ID, USA

**Keywords:** multisensory processing, audiovisual speech perception, McGurk effect, nativism debate, Molyneux's question

When William Molyneux posed his famous thought experiment to the philosopher, John Locke, it was framed to address the transfer of sensory information between touch and vision. Molyneux's Question (MQ), appearing in Altieri (2015) and also Schwenkler (2015), was stated by Locke:

“Suppose a man born blind, and now adult, and taught by his touch to distinguish between a Cube, and a Sphere…, so as to tell, when he felt one and t'other, which is the Cube, which the Sphere. Suppose then the Cube and Sphere placed on a Table, and the Blind Man to be made to see. *Quære*, whether by his sight, before he touched them, he could now distinguish, and tell, which is the Globe, which the Cube.”

-Locke (1694/1979).

I argue in this paper that MQ has far broader ramifications than a thought experiment inquiring whether visual-to-tactile representations are acquired through sensory experience. Specifically, I will respond to Schwenkler (2015) and argue that MQ generalizes, in certain cases, to other modalities such as audition and vision.

Recently, I argued (2015) that an affirmative answer to MQ does not necessarily imply nativism, which is the position that (certain) ideas or representations exist prior to sensory experience. Nativist theories, Schwenkler (2015) and I seem to agree, require additional assumptions before they definitively predict a “Yes” response to MQ. Such a variety of nativism requires sensory information—auditory and tactile in the specific case of MQ—to subsist in a *common amodal code*. To illustrate this point, consider what would be required for a newly sighted individual, already familiar with the form of a sphere and the cube through the sense of touch, to prove capable of identifying them by sight alone. An abstract representation of these shapes must have arisen outside the tactile modality to become immediately accessible to vision^1^.

## Problems with common code theory?

The primary controversy described by Schwenkler (2015) concerns the extent to which MQ generalizes to situations involving other sensory modalities given the veracity of *common code theory*. Take, for instance, the McGurk effect (McGurk and MacDonald, 1976). Akin to MQ, I previously argued that common code theory predicts that newly sighted individuals should experience the McGurk effect—perhaps at a rate similar to typically developing adults (cf. Altieri, 2015; The canonical effect occurs when auditory/ba/is presented with a speaker's face articulating “ga”; listeners, typically hear a fused consonant such as “da” rather than/ba/).

Because the McGurk effect relates sound and vision, whereas MQ relates touch and vision, the problem now becomes: does common code theory requires an affirmative answer to both MQ and Altieri's question? First, I will argue that it should be the case that common code theory predicts an affirmative response, unless it makes one of the following *ad hoc* assumptions referenced by Schwenkler (2015): (1) “it might be thought that low-level spatial and temporal properties are commonly coded, whereas high-level ones like abstract category membership are not,” and furthermore, (2) “…it might be that vision and touch share an innate common code, whereas the connections between these modalities and those of smell and taste have to be learned” (p. 2). Hence, common code theory can be subdivided into different varieties, similar to the way I showed how different varieties of nativism can be specified. While such a sub-division appears logically possible, it nullifies the basis of common code theory inasmuch as it fails to assume that perception, qua recognition and categorization, occurs in a manner that is inherently amodal. As I stated (2015) “… recognition must occur through, or representations must be translated into a common amodal code (e.g., dynamic events or gestures)” (p. 2).

Thus, I argue that while logically possible for common code theory to be construed in such a way that it predicts an affirmative response for MQ but not Altieri's question, this scenario is unlikely. Supposing that sensory information is amodal, then, we have to ask how it could be possible for a newly sighted person to visually identify an object previously only privy to the tactile modality, but not the auditory. How is it that certain brain areas easily share information while others have to develop connections, or stated differently, why should preference be given to one modality over another if perception occurs apart from modality? Nonetheless, Schwenkler (2015) brings up an important point, apparent in point (2) above. Paraphrasing, it seems that one potential problem is that even when considering amodal representations, connections between brain regions would either have to develop, whereas others may subside innately (e.g., tactile-visual). This concern becomes potentially more problematic in individuals with periods of sensory deprivation during development.

It is important to emphasize, however, that the question of what sensory modalities take precedent over others (e.g., whether visual cues are more apt to affect auditory or tactile ones or vice versa) is purely an empirical matter. Future research should benefit from examining the extent to which vision overrides auditory or tactile cues in typically developing, blind, and newly sighted individuals. The visual dominance of the *ventriloquist effect* indicates that visual cues naturally override auditory information (Alais and Burr, 2004). Sensory development also appears to alter auditory-tactile mappings: One study showed that multiple auditory stimulations evoke illusory tactile perceptions in sighted but not congenitally blind subjects (Hotting and Roder, 2004). This brings us to the following conclusion: I propose that if the results of cross-modal studies—using visual-tactile and visual-auditory paradigms—indicate similar performance across newly sighted and normal adults, it would lend converging support for common code theory. Conversely, differences would support either modular (cf. Altieri, 2015) or possibly empiricist theories.

## Author contributions

The author confirms being the sole contributor of this work and approved it for publication.

### Conflict of interest statement

The author declares that the research was conducted in the absence of any commercial or financial relationships that could be construed as a potential conflict of interest.

## Footnotes

^1^Other nativist theories discussed by Altieri (2015) need not make such strong assumptions and therefore can predict a negative response to MQ. One variety assumes the innateness of representations in one modality although robust cross-modal associations can arise out of perceptual experience or learning.
